# Utility of Antinephrin Autoantibody–to–IgG Ratio in Autoimmune Podocytopathies

**DOI:** 10.1016/j.ekir.2026.106465

**Published:** 2026-03-11

**Authors:** Kenichiro Miura, Yoko Shirai, Shigeru Horita, Mayumi Abe, Keiichi Takizawa, Yutaka Harita, Etsuko Tanaka, Kiyohiko Hotta, Saori Nishio, Motoshi Hattori

**Affiliations:** 1 Department of Pediatric Nephrology, Tokyo Women’s Medical University, Tokyo, Japan; 2 Department of Pediatrics, The University of Tokyo, Tokyo, Japan; 3 Department of Pediatrics, Faculty of Medicine, University of Miyazaki, Miyazaki, Japan; 4 Department of Renal and Genitourinary Surgery, Faculty of Medicine, Hokkaido University, Sapporo, Japan; 5 Department of Hemodialysis and Apheresis, Hokkaido University Hospital, Sapporo, Japan; 6 Kidney Disease Research Institute, Tokiwa Foundation, Jyoban Hospital, Iwaki, Fukushima, Japan

**Keywords:** antinephrin autoantibody, false-negative, focal segmental glomerulosclerosis, IgG, nephrotic syndrome, urinary loss

## Introduction

Since the discovery of antinephrin autoantibodies as a cause of minimal change disease,^1^ many studies have demonstrated their association with autoimmune podocytopathies, including minimal change disease and focal segmental glomerulosclerosis (FSGS).^2^, ^3^, ^4^, ^5^ However, because the enzyme-linked immunosorbent assay method and cut-off values are not standardized, the assessment of antinephrin autoantibody positivity remains a matter of debate.^6^^,^^7^ Furthermore, in nephrotic syndrome, autoantibodies may be underestimated because of urinary protein loss.^7^ Therefore, values adjusted for concomitant IgG loss may be more relevant for clinical use.

In this study, we measured the antinephrin autoantibody–to–IgG ratio in patients with steroid-resistant nephrotic syndrome (SRNS) or posttransplant FSGS recurrence who underwent serial measurements of antinephrin autoantibodies (Supplementary Methods, Supplementary Figure S1), and evaluated its clinical utility. This study was approved by the ethics board of Tokyo Women’s Medical University (No. 2021-0184).

## Results

### Patients’ Clinical Characteristics

**Figure 1:**
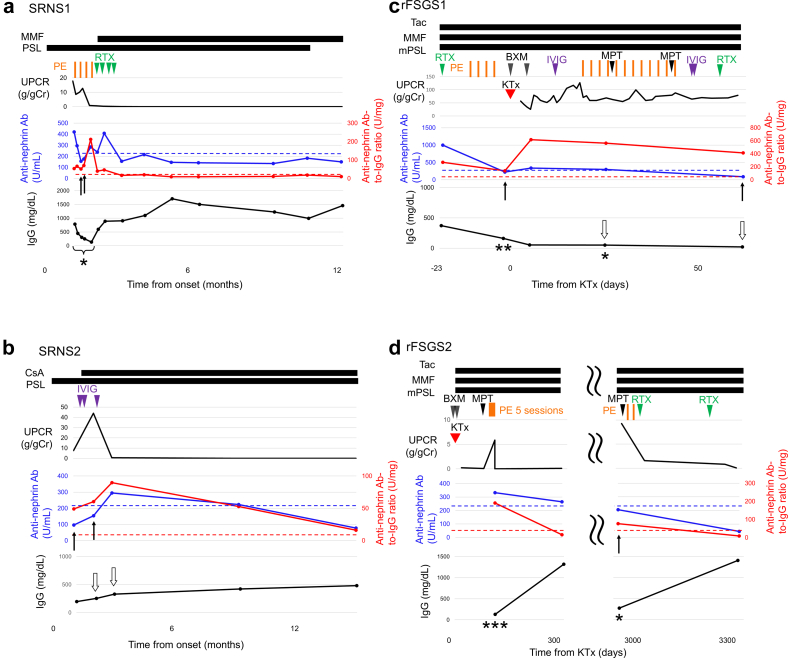
Patients’ clinical courses and serial changes in antinephrin autoantibodies and antinephrin autoantibody–to–IgG ratio. Blue lines represent antinephrin autoantibody levels, whereas red lines represent the antinephrin autoantibody–to–IgG ratio. Dotted lines indicate the respective cut-off values (226 U/ml and 30 U/mg). In all patients, antinephrin autoantibodies fluctuated between positive and negative during nephrotic-range proteinuria, whereas the antinephrin autoantibody–to–IgG ratio was positive at nearly all time points during proteinuria. Notably, all time points at which antinephrin autoantibodies were negative but the antinephrin autoantibody–to–IgG ratio was positive occurred either during nephrotic-range proteinuria or immediately after plasma exchange, and serum IgG levels were very low (black arrows). Although i.v. Ig may have increased the serum IgG levels at 2 time points in patient SRNS2 (white arrows), the actual antinephrin autoantibody–to–IgG ratio would likely have been even higher; therefore, the positive results would not be expected to change at those time points. In patient rFSGS1, serum IgG levels remained extremely low because of heavy proteinuria despite i.v. Ig administration (white arrows), suggesting that i.v. Ig had little effect on the antinephrin autoantibody–to–IgG ratio. Ab, antibody; BXM, basiliximab; CsA, cyclosporine; FSGS, focal segmental glomerulosclerosis; KTx, kidney transplantation; MMF, mycophenolate mofetil: mPSL, methylprednisolone; MPT, methylprednisolone pulse therapy; PE, plasma exchange; PSL, prednisolone; RTX, rituximab; SRNS, steroid-resistant nephrotic syndrome; Tac, tacrolimus; UPCR, urine protein-to-creatinine ratio. ∗Serum samples were obtained before each session of PE. ∗∗Serum samples were obtained 2 days after completion of prophylactic PE and prior to KTx. ∗∗∗Serum samples were obtained ten days after completion of PE.

Four patients were analyzed in this study. Of these, 2 patients had SRNS (SRNS1 and 2, and both achieved complete remission with multiple therapies, including methylprednisolone pulse therapy, cyclosporine, plasma exchange, and rituximab [Figure 1a and b, Supplementary Table S1]). Two patients had posttransplant FSGS recurrence (rFSGS1 and 2, Supplementary Table S2). FSGS recurred on postoperative days 1 and 76 in rFSGS1 and rFSGS2, respectively (Figure 1c and d). One patient (rFSGS1) progressed to graft failure 2 years after transplantation, and the other patient (rFSGS2) achieved complete remission with methylprednisolone pulse therapy, rituximab, and plasma exchange (Figure 1c and d, Supplementary Table S2). All the patients had IgG depositions colocalized with nephrin as demonstrated by native kidney or allograft biopsies (Supplementary Figure S2). Patients SRNS1 and rFSGS1 have been previously reported on, but only antinephrin autoantibodies were measured in those reports.^6^^,^^8^

### Serial Changes in Serum Antinephrin Autoantibody Levels and Serum Antinephrin Autoantibody–To–IgG Ratio

In Figure 1, we show the serial changes in serum antinephrin autoantibody levels and serum antinephrin autoantibody–to–IgG ratio in the patients. In patients SRNS1 and SRNS2, the antinephrin autoantibody fluctuated between negative and positive when nephrotic-range proteinuria was present. In contrast, the antinephrin autoantibody–to–IgG ratio remained consistently positive during periods of proteinuria (Figure 1a and b). In rFSGS1, although antinephrin autoantibodies appeared to have become negative after prophylactic treatment with rituximab and plasma exchange before transplantation, the antinephrin autoantibody–to–IgG ratio remained positive. Consequently, severe recurrence with nephrotic-range proteinuria developed immediately after transplantation (Figure 1c). In rFSGS2, although antinephrin autoantibodies were negative at the time of relapse of proteinuria around postoperative day 3000, the antinephrin autoantibody–to–IgG ratio was positive (Figure 1d). In all patients, time points at which antinephrin autoantibodies were negative but the antinephrin autoantibody–to–IgG ratio was positive occurred either during nephrotic-range proteinuria or immediately after plasma exchange, with extremely low serum IgG levels, as indicated by the black arrows (Figure 1a–d). In patients SRNS1, SRNS2, and rFSGS2, both the antinephrin autoantibody and the antinephrin autoantibody–to–IgG ratio became negative after remission (Figure 1a, b, and d).

### Comparison of Antinephrin Autoantibody and Antinephrin Autoantibody–to–IgG Ratio During Proteinuria and Remission

**Figure 2:**
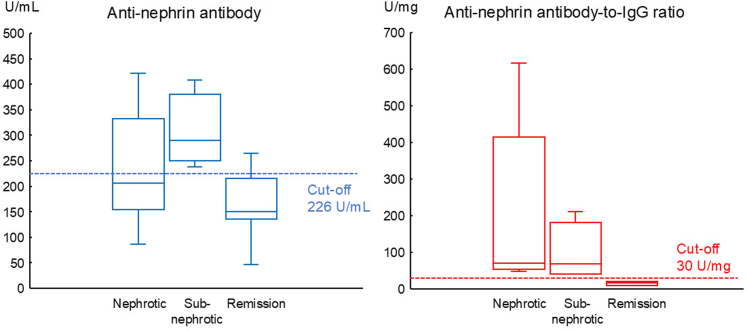
Antinephrin autoantibody levels and antinephrin autoantibody–to–IgG ratio during proteinuria and remission. Antinephrin autoantibody levels varied from positive to negative during periods of nephrotic-range proteinuria, whereas the antinephrin autoantibody–to–IgG ratio was positive at all times during proteinuria. Both the antinephrin autoantibody and the antinephrin autoantibody–to–IgG ratio became negative during remission in most cases.

In Figure 2, we show the antinephrin autoantibody levels and the antinephrin autoantibody–to–IgG ratio during proteinuria and remission. All data points from all patients were analyzed. Antinephrin autoantibody levels varied from positive to negative during periods of nephrotic-range proteinuria, whereas they were positive at all time points during subnephrotic proteinuria. In contrast, the antinephrin autoantibody–to–IgG ratio was positive at all times during episodes of proteinuria, regardless of whether the proteinuria was nephrotic-range or subnephrotic. Both the antinephrin autoantibody and the antinephrin autoantibody–to–IgG ratio were negative at most time points after the patients achieved remission (Figure 2).

## Discussion

Recent studies have increasingly indicated that antinephrin autoantibodies represent one of the pathogenic mechanisms underlying autoimmune podocytopathies, including minimal change disease and FSGS. They are typically positive at disease onset or relapse and become negative after remission, suggesting that they reflect disease activity.^1^, ^2^, ^3^ However, it has been noted that in patients with nephrotic-range proteinuria, antinephrin autoantibodies may become negative because of urinary loss.^7^ In addition, plasma exchange removes circulating autoantibodies as well as IgG. Under these circumstances, the apparent disappearance of autoantibodies does not necessarily indicate suppressed disease activity, and the serum antinephrin autoantibody–to-IgG ratio may more accurately reflect disease status of nephrotic syndrome. In the present study, we investigated the longitudinal changes in serum antinephrin autoantibody titers and antinephrin autoantibody–to–IgG ratios in patients with SRNS and in those with posttransplant FSGS recurrence associated with antinephrin autoantibodies, including patients who underwent plasma exchange.

As a result, in all cases, antinephrin autoantibody titers were often low or negative during periods of nephrotic-range proteinuria, whereas the antinephrin autoantibody–to–IgG ratio remained consistently positive during periods of proteinuria and became negative in remission. In those who underwent plasma exchange, antinephrin autoantibodies often became apparently negative because of the removal of total IgG; however, the antinephrin autoantibody–to–IgG ratio remained positive as long as proteinuria persisted. These findings suggest that the antinephrin autoantibody–to–IgG ratio may reflect the pathogenesis and activity of nephrotic syndrome more accurately than the autoantibody titer itself, although larger studies with more patients are needed.

Many cases with autoimmune podocytopathies exhibiting antinephrin autoantibody titers around the cut-off value have been reported in the literature.^1^^,^^4^ In such cases, assessment should be performed in the context of other clinical information, including nephrin and IgG staining in kidney tissue. Our findings indicate that the degree of proteinuria should also be taken into account when interpreting the presence or absence of antinephrin autoantibodies.

In conclusion, when analyzing antinephrin autoantibodies in patients with nephrotic-range proteinuria, false-negative results due to urinary loss are possible. In addition, there may be cases in which plasma exchange transiently removes antinephrin autoantibodies but does not effectively suppress disease activity. Under these circumstances, the antinephrin autoantibody–to–IgG ratio may provide a more accurate reflection of disease status and activity.

## Disclosure

All the authors declared no competing interests.

## Supplementary Material

Supplementary File (PDF) **Supplementary Methods. Supplementary References. Table S1.** Clinical characteristics of patients with SRNS. **Table S2.** Clinical characteristics of patients with posttransplant FSGS recurrence. **Figure S1.** Distribution of control values of antinephrin autoantibodies and antinephrin autoantibody–to–IgG ratio. **Figure S2.** Representative immunofluorescence images showing double immunostaining for IgG and nephrin observed with structured illumination microscopy.

## Footnotes

Supplementary File (PDF)

**Supplementary Methods.**

**Supplementary References.**
**Table S1.** Clinical characteristics of patients with SRNS. **Table S2.** Clinical characteristics of patients with posttransplant FSGS recurrence. **Figure S1.** Distribution of control values of antinephrin autoantibodies and antinephrin autoantibody–to–IgG ratio. **Figure S2.** Representative immunofluorescence images showing double immunostaining for IgG and nephrin observed with structured illumination microscopy.
