# Supplementary material for: Utility of Antinephrin Autoantibody–to–IgG Ratio in Autoimmune Podocytopathies
Source: Kidney Int Rep. 2026 Mar 11;11(5):106465. doi: 10.1016/j.ekir.2026.106465 (PMC13088958; doi:10.1016/j.ekir.2026.106465)

Supplementary Methods

Sampling

Serum samples were collected longitudinally at multiple points whenever possible, with informed consent obtained from the patients or their guardians. The exact timing of sampling was left to the discretion of each attending physician. The study was approved by the ethics committees of Tokyo Women's Medical University (No. 2021-0184) and each participating institution.

ELISA

Anti-nephrin autoantibodies were measured by ELISA using the recombinant extracellular domain of nephrin derived from human embryonic kidney cells, as previously described[6]. The cut-off value was set at 226 U/mL[6]. Serum IgG was measured using an ELISA kit (Human IgG ELISA Kit, ab195215, Abcam, England). The cut-off value for anti-nephrin autoantibody–to–IgG ratio was set at 30 U/mg, corresponding to the maximum value observed in the control group consisting of membranous nephropathy (n = 13), lupus nephritis (n = 4) and healthy individuals (n = 13). The distribution of control values of anti-nephrin autoantibodies and anti-nephrin autoantibody-to-IgG ratio are shown in Supplementary Figure S1.

Immunofluorescence studies

Immunofluorescence (IF) studies were performed on 2-μm-thick frozen specimens. An anti-nephrin antibody (Anti-Human Nephrin (C) Rabbit IgG Affinity Purify, Immuno-Biological Laboratories, Fujioka, Japan) at a dilution of 1:50 and mouse monoclonal anti-human IgG Fc (ab99757, Abcam, MA, USA) at a dilution of 1:100 were used. As a secondary antibody, goat anti-rabbit secondary antibody, Alexa Fluor 555 (Thermo Fisher Scientific, Waltham, MA) at a dilution of 1:500 and goat anti-mouse secondary antibody, Alexa Fluor 488 (Thermo Fisher Scientific, Waltham, MA) at a dilution of 1:1000 were used. Images were acquired on a ZEISS LSM 900 confocal microscope (Zeiss, Oberkochen, Germany).

All IF images were obtained by the 2-dimensional structured illumination microscopy mode with a Nikon microscope (N-SIM, Nikon), and image reconstruction was carried out using NIS-Elements software (Nikon) on the basis of a previous report with kind support from Dr. Noriko Tokai of the Imaging Core Laboratory (Institute of Medical Science, The University of Tokyo).[2,3,S1]

Definitions

Steroid-resistant nephrotic syndrome (SRNS) was defined as the absence of complete remission within 4 weeks of treatment with prednisolone at the standard dose.[S2] Nephrotic-range proteinuria was defined as urine protein creatinine ratio (UPCR) ≥200 mg/mmol (2 mg/mg) in first morning void

or 24 h urine sample ≥1000 mg/m^2/day corresponding to 3+ or 4+ by urine dipstick.[S2] Complete remission was defined as UPCR (based on first morning void or 24 h urine sample) ≤20 mg/mmol (0.2 mg/mg) or negative or trace dipstick on three or more consecutive occasions.[S2] Subnephrotic proteinuria was defined as proteinuria that did not meet the criteria for nephrotic-range proteinuria or complete remission.

Supplementary References

S1. Gustafsson MG. Surpassing the lateral resolution limit by a factor of two using structured illumination microscopy. J Microsc. 2000;198:82–87. https://doi.org/10.1046/j.1365-2818.2000.00710.x.

S2. Trautmann A, Vivarelli M, Samuel S, Gipson D, Sinha A, Schaefer F, Hui NK, Boyer O, Saleem MA, Feltran L, Müller-Deile J, Becker JU, Cano F, Xu H, Lim YN, Smoyer W, Anochie I, Nakanishi K, Hodson E, Haffner D; International Pediatric Nephrology Association. IPNA clinical practice recommendations for the diagnosis and management of children with steroid-resistant nephrotic syndrome. Pediatr Nephrol. 2020;35:1529–1561. https://doi.org/10.1007/s00467-020-04519-1.

Supplementary Table S1. Clinical characteristics of patients with SRNS

Patients	Sex	Age at onset	Age at kidney biopsy	Treatment	Outcome
SRNS1	M	15	15	PSL, MPT, CsA, MMF, PE, RTX	Complete remission
SRNS2	F	10	10	PSL, CsA	Complete remission

CsA, cyclosporine; MMF, mycophenolate mofetil; MPT, methylprednisolone therapy; PE, plasma exchange; PSL, prednisolone; RTX, rituximab.

Supplementary Table S2. Clinical characteristics of patients with post-transplant FSGS recurrence

Patients	Sex	Age at onset	Age at KTx	Urine output before KTx	SCr at KTx (mg/dL)	Days at post-transplant recurrence	Days at graft biopsy	Treatment for post-transplant recurrence	Graft outcome
rFSGS1	F	1	7	Anuric	8.4	1	21	PE, RTX	Graft failure 2 years after KTx
rFSGS2	M	16	42	Anuric	14.9	76	79	MPT, PE, RTX	Complete remission

KTx, kidney transplantation; MPT, methylprednisolone pulse therapy; PE, plasma exchange; RTX, rituximab; SCr, serum creatinine

Supplementary Figure S1. Distribution of control values of anti-nephrin autoantibodies and anti-nephrin autoantibody-to-IgG ratio.
MN, membranous nephropathy; LN, lupus nephritis; gFSGS, genetic FSGS.

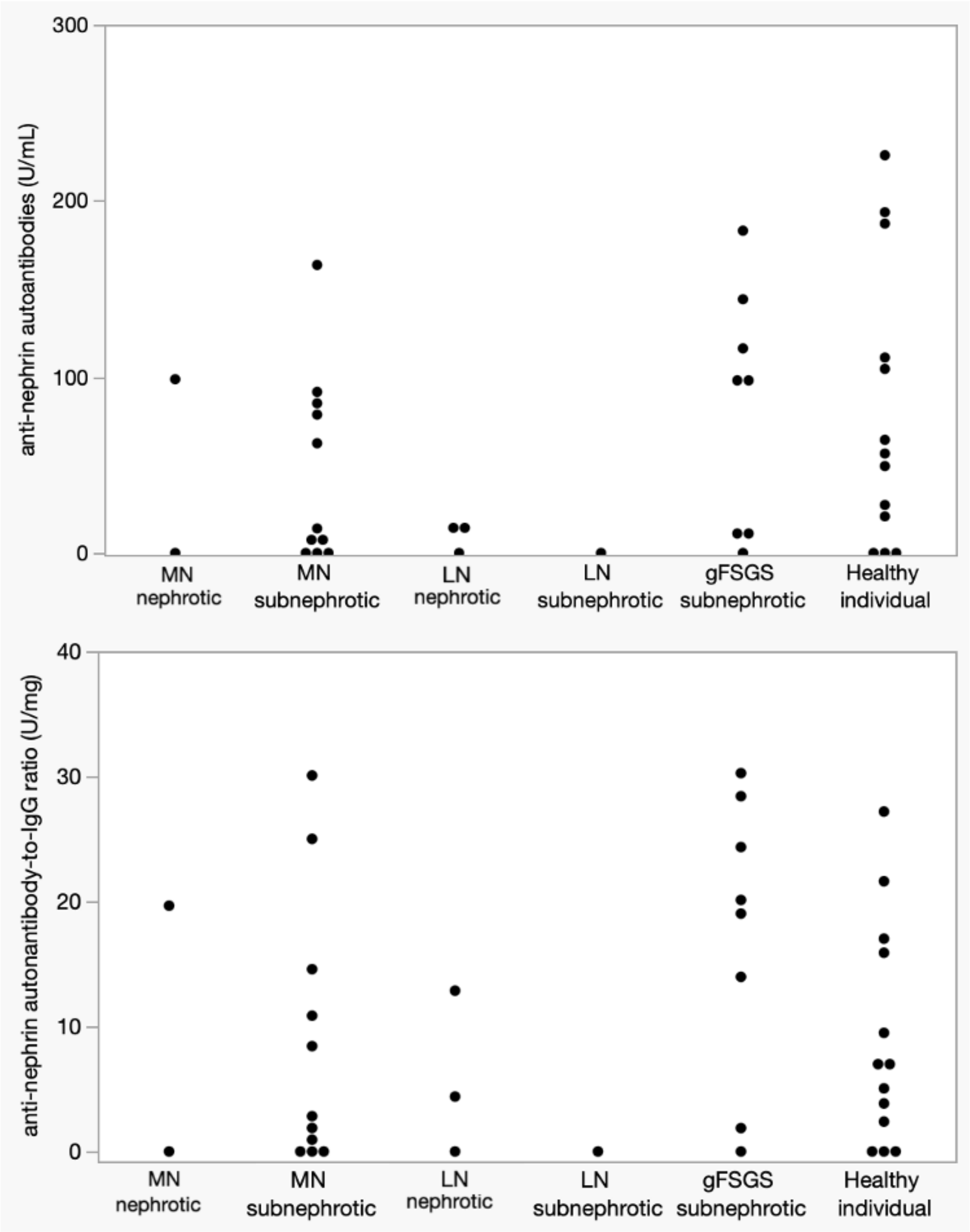

Supplementary Figure S2. Representative immunofluorescence images showing double immunostaining for IgG and nephrin observed with structured illumination microscopy.
All patients showed IgG depositions colocalized with nephrin. In rFSGS2, both IgG deposits that colocalize with nephrin and those that do not are observed.
Scale bar = 20 μm

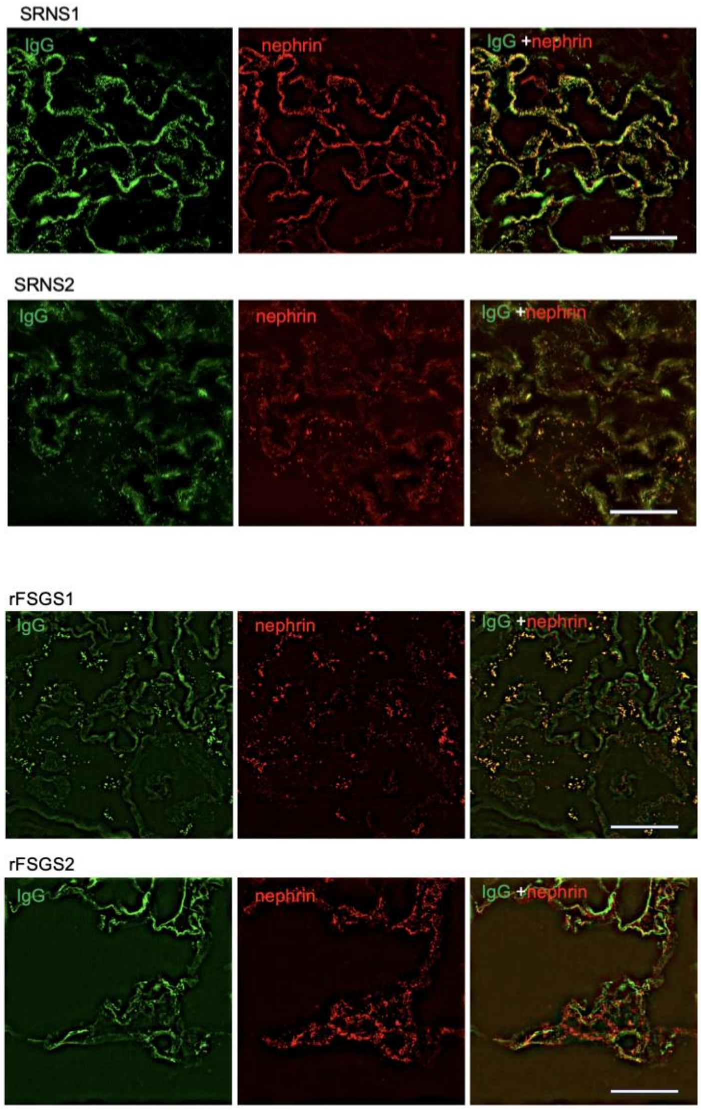

Supplement: Supplementary File (PDF) — Supplementary Methods. Supplementary References. Table S1. Clinical characteristics of patients with SRNS. Table S2. Clinical characteristics of patients with posttransplant FSGS recurrence. Figure S1. Distribution of control values of antinephrin autoantibodies and antinephrin autoantibody–to–IgG ratio. Figure S2. Representative immunofluorescence images showing double immunostaining for IgG and nephrin observed with structured illumination microscopy. [file mmc1.pdf]
